# Role and Contribution of Serological Surveillance in Animals and Exposed Humans to the Study of Zoonotic Influenza Disease Epidemiology: A Scoping Review

**DOI:** 10.3390/pathogens14080739

**Published:** 2025-07-27

**Authors:** Rebecca Badra, Wenqing Zhang, John S. L. Tam, Richard Webby, Sylvie van der Werf, Sergejs Nikisins, Ann Cullinane, Saad Gharaibeh, Richard Njouom, Malik Peiris, Ghazi Kayali, Jean-Michel Heraud

**Affiliations:** 1 Human Link, Dubai Multi Commodities Centre, Dubai P.O. Box 48800, United Arab Emirates; rebecca@human-link.org; 2 Global Influenza Programme, World Health Organization, CH-1211 Geneva, Switzerland; zhangw@who.int (W.Z.); nikisinss@who.int (S.N.); 3 Department of Applied Biology and Chemical Technology, The Hong Kong Polytechnic University, Hong Kong SAR 999077, China; john.sl.tam@connect.polyu.hk; 4 St. Jude Children’s Research Hospital, Memphis, TN 38105, USA; richard.webby@stjude.org; 5 Molecular Genetics of RNA Viruses Unit, Institut Pasteur, F-75015 Paris, France; sylvie.van-der-werf@pasteur.fr; 6 The Irish Equine Centre, Johnstown, Naas, W91 RH93 Kildare, Ireland; acullinane@irishequinecentre.ie; 7 Department of Veterinary Pathology and Public Health, Jordan University of Science and Technology, Irbid 22110, Jordan; saadgh@just.edu.jo; 8 Centre Pasteur du Cameroun, Yaoundé P.O. Box 1274, Cameroon; njouom@pasteur-yaounde.org; 9 School of Public Health, The University of Hong Kong, Hong Kong SAR 999077, China; malik@hku.hk

**Keywords:** zoonotic influenza virus, serological surveillance, wildlife, domestic animals, animal–human interface

## Abstract

**Background:** Zoonotic influenza viruses pose a significant and evolving public health threat. In response to the recent rise in H5N1 cross-species transmission, the World Health Organization (WHO) R&D Blueprint for Epidemics consultations have prioritized strengthening surveillance, candidate vaccines, diagnostics, and pandemic preparedness. Serological surveillance plays a pivotal role by providing insights into the prevalence and transmission dynamics of influenza viruses. **Objective:** This scoping review aimed to map the global research landscape on serological surveillance of zoonotic influenza in animals and exposed humans between 2017, the date of the last WHO public health research agenda for influenza review, and 2024, as well as to identify methodological advancements. **Methods:** Following PRISMA-ScR guidelines, we searched PubMed for English-language peer-reviewed articles published between January 2017 and March 2024. Studies were included if they reported serological surveillance in wild or domestic animals or occupationally exposed human populations, or novel methodologies and their technical limitations and implementation challenges. **Results:** Out of 7490 screened records, 90 studies from 33 countries, covering 25 animal species, were included. Seroprevalence studies were in domestic poultry and swine. Surveillance in companion animals, wild mammals, and at the human–animal interface was limited. Emerging serological methods included multiplex and nanobody-based assays, though implementation barriers remain. **Conclusions:** The review is limited by its restriction to one database and English-language articles, lack of quality appraisal, and significant heterogeneity among the included studies. Serological surveillance is a critical but underutilized tool in zoonotic influenza monitoring. Greater integration of serological surveillance into One Health frameworks, especially in high-risk regions and populations, is needed to support early detection and pandemic preparedness.

## 1. Introduction

Zoonotic influenza viruses represent a continuous and evolving public health threat given their potential to mutate and reassort, creating new strains that can cross the species barrier and cause widespread outbreaks in humans [1]. Understanding the epidemiology and dynamics of these viruses in animal populations is crucial for mitigating the risk of human infection and for developing effective surveillance and response strategies [2].

Serological surveillance plays a pivotal role in this context, providing insights into the prevalence and transmission dynamics of influenza viruses in both animal reservoirs and exposed human populations [3,4]. It is increasingly being recognized as a valuable tool that could complement traditional passive disease surveillance [5]. Serological surveillance allows for the detection and quantification of antibodies against specific influenza viruses, providing insights into past infections, immune responses, and potential exposure risks [6]. It can detect symptomatic and asymptomatic infections that can otherwise go unnoticed and undetected by traditional surveillance systems [3]. Serological data help elucidate patterns of viral circulation, prevalence rates in different species, and the potential for zoonotic transmission [7]. In animals, serological surveillance can reveal the presence of influenza viruses in wildlife, domestic animals, and livestock, facilitating early detection of new strains that might pose a risk to humans, informing the development of targeted monitoring and intervention strategies. It is useful for detecting asymptomatic and mild infections and changes in disease patterns in animal populations [3]. By identifying exposed and infected animals, serological surveillance can inform the implementation of biosecurity measures to prevent the spread of zoonotic diseases [8]. In exposed human populations, serological testing allows for the identification of past infections, the assessment of immunity levels in a population, and understanding some disease transmission dynamics [5]. Serological surveillance is considered the gold standard for measuring population immunity [9] and generated data can inform vaccination strategies, risk communication, and resource allocation during outbreak responses.

Serological surveillance can be focused on a single pathogen but can also use multiplexing techniques and assays, allowing for the simultaneous detection of a batch of pathogens at the same time in a single reaction. These data could be coupled with other data computational techniques to draw a picture of the distribution of antibodies and infectious diseases [10]. Antigen multiplexing and advances in antigen-level multiplex serology have also been used to differentiate between immunity induced by vaccination versus natural infection [11]. Furthermore, antibody detection could be achieved with small sample volume and a variety of samples can be used [12,13]. Despite the important insights provided by serological surveillance, variability in testing methods, differences in study design, and the potential for cross-reactivity can complicate data interpretation.

The World Health Organization (WHO) Global Influenza Programme developed the WHO public health research agenda for influenza in 2009, highlighting the importance of research in influenza prevention and control to enhance global knowledge, preparedness, and response capabilities related to influenza, and to fill the gaps in understanding the biology, transmission, and epidemiology of influenza viruses and emerging strains with pandemic potential. The agenda was reviewed in 2010–2011 and then again in 2017 to guide researchers and outline directions and priority areas for research for the next 5–10 years. This agenda recommended research to reduce the risk of emergence of a pandemic influenza, its amplification in farmed animals, and its transmission to humans, and focused on improved surveillance and detection of emergent influenza A viruses (IAV) with zoonotic or pandemic potential and serological tests for zoonotic IAV in humans. Since 2017, important research studies have been conducted and major changes have occurred to the global health landscape, impacted mainly by the COVID-19 pandemic. Therefore, there is a need to review serological surveillance studies from the past eight years across different geographic regions and animal species and to identify the new methodologies developed.

The widespread transmission of HPAI H5N1 across wild birds, domestic animals, cattle, and recent human infections has heightened global health concerns. In response, WHO R&D Blueprint for Epidemics held a global consultation to set priorities for containing and mitigating potential pandemic H5N1 outbreaks [14].

This scoping review was conducted to map the available literature on serological surveillance of influenza viruses in domestic animals, wild animals, and at the animal–human interface globally between 2017, the date of the last influenza research agenda review, and 2024, and to evaluate the contribution of serological surveillance in studying the epidemiology of zoonotic influenza viruses. This review also describes novel serological methodologies and their technical limitations and implementation challenges.

## 2. Materials and Methods

### 2.1. Search Strategy

**Figure 1:**
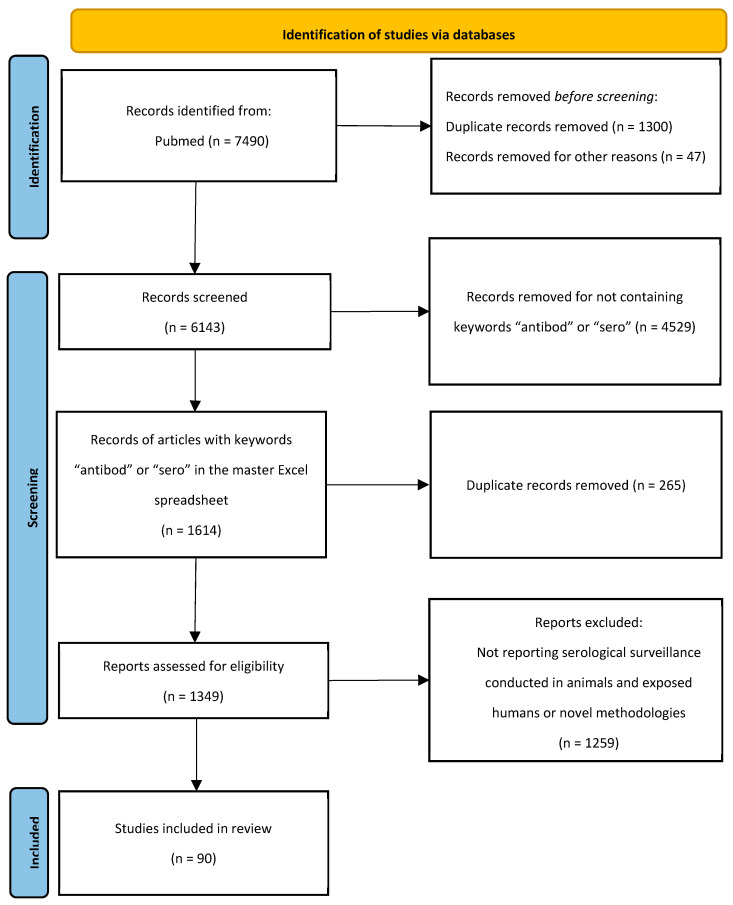
PRISMA-ScR flow diagram of the screening and selection of studies for this scoping review.

This scoping review was reported according to the Preferred Reporting Items for Systematic Reviews and Meta-Analyses extension for Scoping Reviews (PRISMA-ScR). It aimed to map the scope and characteristics of published studies on serological surveillance of zoonotic influenza viruses in animals and humans. This study did not require institutional review board approval. We searched PubMed for peer-reviewed English publications using the search terms “avian influenza”, “swine influenza”, and “zoonotic influenza viruses” from 2017 until 2024 (Figure 1). In total, 7490 records were exported to an Endnote X8 (Endnote, Berkley, CA, USA) library. Duplicates and papers published after 31 March 2024 were removed, yielding 6143 publications retained and exported to a master Excel (Version 2408) spreadsheet (Microsoft, Redmond, WA, USA). The following data were extracted from each included study: publication year, country, and category. To select the papers that discussed serological surveillance conducted in animals and exposed humans, in addition to novel methodologies for serological surveillance, we searched for the keywords “antibod” and “sero” in the master Excel spreadsheet. The search yielded 1097 records for “antibod” and 517 records for “sero” terms. Duplicates were removed and 1349 manuscripts were retained. Those records were exported to a new Excel sheet. Titles and abstracts were reviewed thoroughly and were independently screened by two researchers to select only the papers discussing serological surveillance conducted in animals and exposed humans in addition to novel methodologies. Any disagreements between reviewers were resolved through discussion and a consensus approach; if consensus could not be reached, a senior scientist with expertise in zoonotic influenza adjudicated the decision.

### 2.2. Eligibility Criteria

The included publications met the following eligibility criteria: (1) published in English; (2) publications reporting serological surveillance conducted in animals and exposed humans in addition to novel methodologies for the detection of zoonotic influenza viruses; and (3) publications published in the period from 2017 to end of March 2024. The start date of 2017 was chosen because it aligns with the 2017 update of the WHO public health research agenda for influenza and the timeframe of the current WHO review, which sets updated research priorities for the next 5–10 years; this ensures that the review reflects research conducted in line with those global priorities. Publications were excluded for the following reasons: (1) not published in the English language; (2) duplicates, (3) published after 31 March 2024; or (4) records not reporting serological surveillance conducted in animals and exposed humans or novel methodologies for detection of zoonotic influenza viruses. Studies without full-text access were included. No geographic restrictions were applied during the search. Studies using multiplex serological assays were included if they reported influenza-specific antibody results relevant to the scope of the review.

### 2.3. Data Collection Process

A standardized data extraction form was designed by the study team based on key variables relevant to serological surveillance, including study location, species, virus subtype, assay types, sample size, seroprevalence rates, and any reported technical or operational limitations. The form was piloted on a subset of 10 studies to test its clarity, completeness, and usability, and refinements were made before full data extraction began. Two researchers independently extracted data from the 90 retained peer-reviewed publications. Discrepancies were resolved through discussion and consensus, and all entries were reviewed by a senior scientist. Data were extracted on serological surveillance conducted in domestic animals, wild animals, and at the animal–human interface, as well as the contribution to evaluating the zoonotic influenza disease burden in exposed human populations and novel methodologies with their technical limitations and implementation challenges.

### 2.4. Synthesis of Results

A narrative summary of the characteristics of the selected articles was conducted. Findings were organized by host species (domestic animals, wild animals, and exposed humans), virus subtypes, and surveillance context. Key variables such as seroprevalence rates, sample sizes, geographic locations, and assay types were tabulated and described. The synthesis also highlighted emerging themes, including evidence of interspecies transmission, use of novel serological methods, and geographic gaps in surveillance.

This review was designed as a scoping review due to the heterogeneity in study designs, objectives, diagnostic methods, and outcome reporting among the included studies. No quantitative analysis or meta-analysis was performed. A formal quality appraisal was not performed, as the objective was to explore the breadth and nature of available evidence rather than assess study validity or effect sizes. No visualization was developed (world map, timeline, comparison charts) due to the heterogeneity and complexity of data.

## 3. Findings

A total of 7490 papers were identified via the database search. After screening and exclusion using the pre-defined criteria, 90 research papers were included in this review. The selection was confirmed by a senior scientist with extensive expertise in public health and more than 10 years of experience in zoonotic influenza. The full screening and selection process is illustrated in the PRISMA flow diagram (Figure 1).

### 3.1. Serological Surveillance of Zoonotic Influenza Viruses in Domestic Animal Husbandry

#### 3.1.1. Serological Surveillance of Avian Influenza Virus in Domestic Birds

Serological surveillance studies of avian influenza in birds are summarized in Table 1. In the last eight years, a few peer-reviewed papers have reported studies and findings of serological surveillance of IAV. One study on 359 backyard chicken samples collected from Al-Ahsa Eastern Region in Saudi Arabia between 2015 and 2017 reported 12.7% IAV seroprevalence [15]. One study on 947 free-range and household duck samples collected from wetlands in Bangladesh reported high IAV seroprevalence of 63.8% with eight H5N1 outbreaks in poultry reported during the same period, suggesting that the household ducks might be a source of avian influenza virus (AIV) infection for poultry [16]. In two regions in Bangladesh, a study showed that ducks raised on backyard farms exhibited higher H5 antibody prevalence than in-contact backyard chickens and layer chickens had higher H5 antibody prevalence than broiler chickens. H9 antibody prevalence was similar in backyard chickens and ducks, and higher than that in layers and broilers, suggesting high AIV circulation in live bird markets [17]. Between February and April 2017 in Bangladesh, a study showed 9.4% and 5.7% seropositivity rates for H5 and H9 in asymptomatic broiler flocks and 3% and 22% seropositivity rates for H5 and H9 in asymptomatic layer flocks. Seropositivity was associated with visits of workers from other commercial chicken farms and exposure to backyard ducks [18]. One study, published in 2022, on 281 household ducks sampled from Chattogram in Bangladesh reported AIV seroprevalence of 57.7% with H5 and H9 subtype seroprevalence of 31.5% and 23.9%, respectively. [19]. These results highlighted the continuous circulation of AIV in this region. In Brazil, one study was conducted to assess avian influenza viral activity in backyard poultry raised around migratory bird sites in 2016 and 2019 and showed that, while H5 and H7 AI subtypes were not detected, the circulation of low pathogenic AIV strains in backyard poultry can be a potential infection source for other birds [20]. In March 2019, a study on 245 duck samples from Purbalingga in Indonesia showed 54.7% H5 seroprevalence rate and revealed that the system of nomadic duck farming might be more susceptible to acquire H5 infection than the system of intensive farming [21]. In 2019 in Polynesia, one study reported 46% seropositivity of AIV among 135 domestic chickens as well as the isolation of a new H6N1 low pathogenic virus, highlighting the active circulation of AIV [22]. One study aiming at determining the seroprevalence of major infectious diseases in backyard poultry in Morocco performed on 712 sera samples collected between February 2021 and June 2022 showed that the seroprevalence of H9N2 was 63.5%, suggesting that backyard chicken flocks and rural markets could play the role of reservoirs for poultry pathogens, which pose an important risk to the commercial poultry sector [23]. One study published in 2023 to explore the correlation between high mortality rates among broiler flocks and H9N2 infection in Tripoli, Libya, showed high seroprevalence of AI in areas suffering from a high mortality rate and areas with a low mortality rate, around 50% of which were of the H9N2 subtype, suggesting that H9N2 was not by itself the main cause for the high mortality rate but other subtypes of AI might be present in the studied areas [24]. One study, conducted in chicken in the Eastern Cape Province of South Africa, showed 1.8% seroprevalence of AIV in the province. Hemagglutination inhibition testing showed that all detected antibodies are H6-specific, providing evidence of exposure of village chickens to chicken diseases [25]. The annual report on surveillance of AIV in poultry and wild birds in Member States of the European Union in 2018 reported that 43 poultry establishments were seropositive for H5 and two were seropositive for H7 out of 18,596 establishments [26]. The 2019 annual report showed that 87 poultry establishments were seropositive for H5 and 22 were seropositive for H7 out of 24,419 [27]. The 2020 report showed that 46 poultry establishments were seropositive for H5 and seven were seropositive for H7 out of 24,768 [28]. The 2021 report showed that 27 poultry establishments were seropositive for H5 and four were seropositive for H7 out of 24,290 poultry establishments [29]. Finally, another report summarized surveillance activities conducted in 2022 in European Union member states and showed that 15 poultry facilities were seropositive for H5 out of 18,490 [30]. In all the reports, the highest seropositivity rates were reported in facilities or farms raising breeding geese and waterfowl game birds.

#### 3.1.2. Serological Surveillance of Avian Influenza Virus in Domestic Mammals

Serological surveillance studies of avian influenza in domestic mammals are summarized in Table 2. In southwestern France, following outbreaks in domestic poultry due to highly pathogenic avian influenza (HPAI) A/H5N8 viruses belonging to the A/goose/Guandong/1/1996 (Gs/Gd) lineage, clade 2.3.4.4b, a serological analysis was conducted on 10 pig herds and showed that one backyard pig had antibodies against H5 viruses from clade 2.3.4.4b after close contact with infected domestic ducks, highlighting the important role of biosecurity measures in poultry and pig farms [31]. One surveillance study on 1636 swine samples collected from a slaughterhouse in Dakar in Senegal from September 2018 to December 2019 reported 83.5% seroprevalence of antibodies against either H9N2, H5N1, H7N7, or H5N2, with H7N7 (54.3%) and H9N2 (53.6%) being the dominant avian subtypes [32]. These results highlighted the potential emergence of new variants given the cocirculation of various influenza virus subtypes in Western Africa. A study on 67 pig samples collected from a free-ranging farm in Rome in 2021/2022 after direct contact with poultry infected with HPAI H5N1 reported 73% H5N1 seropositivity in swine, highlighting the importance of enhancing biosecurity measures and effective separation in multispecies farms [33]. Samples form 347 healthy farmed minks and 195 healthy farmed foxes were collected between December 2016 and November 2017 from Eastern China and analyzed, showing 6.6% and 96.2% seropositivity rates for H7 and H9 in mink samples and 16.4% and 10.3% seropositivity rates for H7 and H9 in fox samples, respectively. Additionally, antibodies against both H7 and H9 were detected in some samples, highlighting the spread and cocirculation of H7 and H9 in farmed minks and foxes [34]. A study from Italy reported an outbreak of HPAI H5N1 of clade 2.3.4.4b in April 2023 in a backyard poultry with T271A mammalian adaptive mutation in the PB2 protein. While no human transmission was reported in exposed individuals, seroconversion was detected in five asymptomatic domestic dogs and one cat living on the premises, highlighting the importance of monitoring H5N1 infections in mammalian pets [35]. One study on 195 dromedary camels, sheep, goats, dogs, and cats collected from Eastern Saudi Arabia between September 2018 and March 2019 reported an overall IAV seropositivity rate of 4% among unvaccinated dogs, indicating natural exposure history to IAV [36]. A cross-sectional study conducted in Spain from March 2022 to March 2023 on 183 stray cat samples reported a 2.2% seropositivity rate for anti-influenza A antibodies [37]. This low seropositivity rate suggests that cats did not play a significant role in the transmission of IAV in the regions studied, even though these areas had positive AIV cases in wild birds. In Chiang Mai, Thailand, a cross-sectional study on 237 samples of backyard pigs collected across six districts between September 2016 and February 2017 reported the absence of antibodies against IAV, suggesting low interspecies transmission of IAV in this region [38].

#### 3.1.3. Serological Surveillance of Swine Influenza Virus in Swine and Pigs

Serological surveillance studies of swine influenza virus (IAV-S) in domestic animals are summarized in Table 3. A study conducted in pigs in Africa showed that 32% of the sera collected from pigs in Zambia in 2011 had neutralizing antibodies against human seasonal A(H1N1)pdm09 virus compared to less than 5.3% of sera collected from 2012 to 2018. One H3N2 IAV closely related to human seasonal H3N2 lineage and eight H1N1 IAV strains closely related to A(H1N1)pdm09 virus were isolated form nasal swab samples, providing evidence of a reverse zoonotic transmission from humans to pigs [39]. One study, conducted on 649 pig samples collected from Banten and West Java provinces in Indonesia between February 2016 and November 2017, showed 26% seropositivity of IAV-S, with areas of high pig and poultry farm density identified as risk factors for IAV-S seropositivity [40]. A cross-sectional study on 600 pig samples collected from 62 swine farms in Burkina Faso between 2016 and 2017 reported 6.8% seropositivity to human seasonal A(H1N1)pdm09 virus, providing evidence of potential reverse zoonosis transmission from humans to pigs [41]. One study, on 40,343 swine samples collected from unvaccinated swine between 2016 and 2021 from 17 Chinese regions, reported an overall 48.8% IAV-S seropositivity with 24.7%, 7.9%, and 0.1% seropositivity rates for Eurasian avian-like H1N1, pandemic H1N1, and H3N2 IAV-S subtypes, respectively [42]. One serological study on 1631 unvaccinated pig samples collected between 2017 and 2019 from commercial herds in Brazil reported 75% IAV seropositivity, with the detection of IAV-S (H3N2 and pandemic H1N1 subtypes), suggesting that swine H1N1 is not the most prevalent subtype and that a pattern of alternating outbreaks or prevalence between swine H3N2 and H1N1 exists in Brazilian swine herds [43]. A cross-sectional study on 233 sow samples from breeding farms from southern Brazil reported that the swine pandemic H1N1 was the most prevalent subtype with cocirculation of IAV-S H1N1, H1N2, and H3N2 in the pig farms. The presence of bird-proof nets and the use of acclimatization units for gilts were correlated with a lower IAV seroprevalence [44]. In Greece, a study on 1416 pig samples collected from March 2019 to April 2023 from commercial pig farms showed 54% IAV-S seropositivity in vaccinated pigs with inactivated vaccines against IAV-S (H1N1, H3N2, and H1N2), and 23% seropositivity in unvaccinated pig farms, providing evidence of circulation of the virus in both vaccinated and unvaccinated pigs. The study also showed lower levels of maternally derived antibodies in unvaccinated pigs compared to vaccinated pigs, highlighting the importance of vaccination programs [45]. A study conducted between March and August 2023 to assess the prevalence of swine viral diseases in 222 pigs from 69 backyard farms with low biosecurity in Serbia showed the absence of swine influenza seroconversion [46]. A study conducted in Argentina on 68 backyard and small producers showed that 80% of the farms had antibodies against swine pandemic H1 and 11% of the producers were positive to swine influenza H3N2. Another study, conducted on 601 blood samples from five intensive farms and 361 blood samples from 56 extensive farms, showed that 24.13% of samples from intensive herds were positive for IAV-S, with the highest prevalence detected in sow and weaning piglets. Risk factors associated with the occurrence of IAV-S included exposure to recently introduced animals [47]. A systematic review conducted to understand the molecular and serological prevalence of IAV in backyard swine populations globally until 2011 showed that more than 44% reported human-to-swine transmission of IAV, with the human-origin A(H1N1)pdm09 virus clade 1A.3.3.2 being the most frequently detected IAV subtype. The transmission of AI between species has been correlated with human–swine and avian–swine interactions in backyard swine population [48]. A serological study conducted in the southeast region of Brazil on 21 pig herds showed that the seroprevalence of IAV-S gradually increased throughout the raising phases from 29.2% to 51.8%, with IAV-S seropositivity in nursery pigs associated with the presence of cough episodes in growing pigs [49].

#### 3.1.4. Serological Surveillance of Canine Influenza Virus in Domestic Animal Husbandry

In Hong Kong, one study on 555 companion dogs and 182 shelter dogs from 2015 to 2018 reported a 0.9% seropositivity rate for canine influenza virus (H3N8 or H3N2 subtypes) and 7.5% seropositivity rate for human influenza virus (A(H1N1)pdm09 or H3N2 subtypes), highlighting the potential interspecies transmission of influenza viruses between people and their companion animals [50]. One study analyzed 496 dog samples collected from Poland in 2016/2017 and reported 7.2% IAV seropositivity with 1.4%, 4.2%, and 1.6% seropositivity rates for equine H3N8, swine H3N2, and swine H1N1pdm subtypes, respectively, highlighting the importance of surveillance of IAVs in dogs [51]. One study on 3579 healthy and with respiratory symptoms pet dogs from 27 Chinese provinces between January 2017 and December 2018 was conducted and reported a 14.7% seropositivity rate for canine influenza virus (H3N2 subtype) [52]. A second study was conducted on 800 stray dog samples between January and May 2019 in Shanghai and reported 17.6% seropositivity for canine influenza virus (H3N2 subtype) [53].

#### 3.1.5. Serological Surveillance of Influenza D Virus in Domestic Animal Husbandry

In Ireland, a study reported 94.6% influenza D virus (IDV) seroprevalence in 1219 healthy cattle samples collected in January 2017 from slaughterhouses, 64.9% IDV seroprevalence in 1183 cattle samples collected between 2016 and early 2017 for the diagnosis of bovine respiratory disease, and only 4.5% from 288 sheep and 5.8% from 377 pigs collected previously for routine general diagnostic testing, highlighting the widespread presence of IDV among Irish cattle [54]. Another study assessed the seropositivity of IDV among domestic ruminants and swine in West and East Africa from 2017 to 2020 by testing 3381 domestic ruminants and swine samples and reported an overall 6.9% IDV seroprevalence in cattle and low seropositivity among sheep and goats, showing that IDV is still circulating in Africa, with variations in seropositivity among countries and species [55]. Moreover, a study aimed to assess the epidemiologic situation of IDV in Swedish dairy farms by testing 461 and 338 bulk tank milk samples from Swedish dairy farms in 2019 and 2020 and reported 32% and 40% IDV seropositivity in 2019 and 2020, respectively, suggesting the presence of IDV in Swedish dairy herds [56]. In France, a study conducted on 2090 domestic pig and 644 wild boars samples collected from 2009 to 2018 to assess the circulation of IDV in these species confirmed that pigs and wild boars have been exposed to IDV with seropositivity rates of 73.3% and 0.5%, respectively [57].

### 3.2. Serological Surveillance of Zoonotic Influenza Viruses in Wild Animals

#### 3.2.1. Serological Surveillance of Zoonotic Influenza Virus in Wild Birds

Between 2016 and 2018, one study, on 183 eiders samples from three Danish colonies and 427 pink-footed geese samples from Central Norway, reported an overall AIV seroprevalence of 55% and 47% in eiders and pink-footed geese, respectively, with up to 17% seropositivity for H5 and/or H7 subtypes, highlighting the potential spillover risk to humans [58]. One study on seabird samples collected in 2017 from the Arctic area reported the presence of antibodies against AIV in black-legged kittiwake samples and glaucous gull samples, providing evidence of exposure to AIV [59]. Between December 2017 and August 2019, one serosurveillance study on 807 samples collected from non-vaccinated commercial and backyard poultry and captive wild birds in Pakistan reported an overall AIV seroprevalence of 67.4% and identification of H7 and H9 subtypes, highlighting the importance of wild bird surveillance to avoid spillovers [60]. In China, between January 2017 and December 2019, a study on 1705 collected wild ducks’ eggs showed that the prevalence of antibodies against H1, H3, H5, and H7 subtypes was 12.3%, 8.1%, 2.0%, and 3.5%, respectively [61]. In Trinidad and Tobago, one study published in 2018 on 38 samples collected from wild birds, reported that three wild birds had antibodies against H5, underscoring the importance of continuous surveillance of wild and domestic birds [62]. One study on 166 black-bellied whistling duck samples collected in February 2018 and 2019 from coastal Louisiana in USA reported 10% IAV seroprevalence, with isolation of one H10N7 subtype [63]. One study on 115 captured nestling ibis samples collected in 2020/2021 from South Florida in USA showed 95% AIV seropositivity, with the continuous presence of maternal antibodies [64]. Another study, published in 2021 on a Central Iran landfill, reported 12.6% AIV seroprevalence out of 150 collected samples from wild birds, highlighting the risk of spillover to landfill workers [65]. A study conducted in winter 2020–2021 on trapped migratory aquatic birds in northeast Italy showed 70.3% IAV seroprevalence in Eurasian teal and 77.8% in Eurasian wigeon. All HI-positive Eurasian teals displayed seroconversion for two HPAI H5 viruses belonging to clade 2.3.4.4b (H5N8/2020 and H5N5/2016), suggesting that Eurasian teal and Eurasian wigeon can play the role of long-distance vectors for HPAI viruses [66]. A study was conducted in Bangladesh on 3585 wild and domestic birds and showed that AIV seroprevalence in both resident and migratory wild birds is not high compared to wild birds from regions, providing evidence that wild birds may not be the origin of AIV infections in the local poultry sector in Bangladesh but could be infected by spillover from poultry and contribute to the spread of the virus [67].

#### 3.2.2. Serological Surveillance of Zoonotic Influenza Virus in Wild Mammals

In Alaska, USA, one study, on samples collected from three loon species collected between 2008 and 2017, showed the detection of IAV antibodies among the collected samples, providing evidence of circulating IAV in loon species in Alaska [68]. In Brazil, one study on 61 wild boar samples collected between October 2017 and November 2018 reported 9.8% IAV seropositivity [69]. In Nigeria, a cross-sectional study on 184 dromedary samples collected from four local areas that border Niger Republic, published in 2022, showed an overall 10.33% IAV seroprevalence, highlighting the continuous circulation of IAV among animals in bordering areas [70]. In the Netherlands, one study on 405 dead wild carnivore samples collected from 2020 to 2022 reported 20% HPAI H5 seropositivity [71]. Some studies explored the seroprevalence of AIV in seals. One study on 45 healthy seal samples collected in 2021 reported the absence of antibodies against H5 [72]. Then, a retrospective study on 59 gray and 266 harbor seal samples collected from 2020 to 2023 did not detect antibodies against AIV [73]. In Thailand, one study on 672 cynomolgus macaque samples collected from March to November 2019 reported 19% IAV seroprevalence, with antibodies against AIV H1, H2, H3, H9, and human H1 (NP-045) viruses [74].

#### 3.2.3. Serological Surveillance of Swine Influenza Virus

In southern Japan, one serological study on wild boar samples collected from 2014 to 2017 reported 27.1% H1N1 IAV-S seropositivity. Some 1.7% were positive for both swine H1N2 and H3N2 viruses, highlighting the important role that wild boars could play in the dynamics of H1N1 in the wild [75]. One study on 1396 wild boar samples collected during two sampling periods (February 2014–September 2016 and October 2016–February 2019) from Bavaria in Germany reported a 9.5% IAV seroprevalence rate in the first period and 5.2% in the second period. The most prevalent antibodies were against IAV-S H1N1. Antibodies against swine H1N2, swine H1 reassortant viruses circulating in domestic pigs in Bavaria, and against AIV H5N8 were detected, highlighting the exposure of wild boar to IAV of diverse origin [76].

### 3.3. Serological Surveillance at the Animal–Human Interface

#### 3.3.1. Serological Surveillance at the Bird–Human Interface

One cohort study on 2402 samples collected from Egypt between August 2015 and March 2019 from backyard poultry-exposed people reported 11% H9 seroprevalence, low H5N1 seroprevalence, and no antibodies against H5N8 [77]. In Rawalpindi, Pakistan, a cross-sectional study on 332 poultry professionals between December 2016 and May 2017 showed 50.3% H9 seroprevalence with 100%, 83.3%, 52.4%, 45.5%, and 38.5% seroprevalence rates among laboratory staff, vaccinators, butchers, farm workers, and veterinarians, respectively, highlighting high H9 seroprevalence among poultry professionals [78]. In the Ashanti region in Ghana, one study on 1200 chicken and 102 farmer samples collected from April 2016 to February 2017 reported no detection of antibodies against AIV [79]. Between 2016 and 2017, in Gyeonggi, Korea, a serosurvey on 870 samples collected from poultry farmers who possibly contacted poultry infected with H5N6 did not detect antibodies against H5N6, highlighting that H5N6 did not transmit to humans [80]. A study conducted in 2017 in Lebanon on 69 workers from a farm with H9N2 infections in chickens reported the absence of antibodies against H5N1 and H9N2 [81].

#### 3.3.2. Serological Surveillance at the Mammalian–Human Interface

A cohort study conducted in China on 658 participants between March 2015 and March 2017 reported a 31.5% seropositivity rate for IAV-S (H1N1 or H3N2 subtypes) among both swine-exposed and non-exposed individuals [82]. This finding suggests that exposure to swine did not significantly increase the risk of seroconversion. The observed seropositivity might be explained by the cross-reactivity of swine H3N2 with the seasonal H3N2 virus in humans. A cross-sectional study in Ghana from April 2016 to February 2017 reported a 3.2% seropositivity rate in swine for human H1N1pdm09 and H3N2 [83]. In KwaZulu-Natal Province, South Africa, one study on 84 workers and 51 swine samples collected from February to October 2018 reported high antibody levels against IAV-S (H1N1 and H3N2 subtypes) in workers sera, suggesting the presence of IAV-S in swine farms in South Africa [84]. Following an H7N2 outbreak in cats at a municipal animal shelter in New York City, a cross-sectional study on 121 shelter workers between 25 January and 8 February 2017 from Manhattan and Brooklyn was conducted and reported a 0.8% seropositivity rate for H7N2, suggesting transmission of this virus from cats to humans [85].

### 3.4. Novel Methodologies, Technical Limitations, and Implementation Challenges

Novel methodologies developed during the last eight years for the serological detection of antibodies against influenza viruses are summarized in Table 4. Multiple Enzyme-Linked Immunosorbent Assay (ELISA) methods, including single and tetrameric M2e peptide ELISA assays for avian influenza, MAb-based assays for influenza D virus, competitive ELISAs for H7 antibodies in various birds, recombinant HA1 protein-based ELISA for H5, single-domain antibody assays for swine influenza virus antibodies, novel nanobody-based competitive ELISAs for IAV, and an epitope-blocking ELISA for the detection of H5 antibodies in chicken sera, have been developed, demonstrating high sensitivity, specificity, and potential for widespread use in monitoring and diagnosing these viruses across different species [86,87,88,89,90,91,92,93]. A variety of advanced serological assays, including duplex xMAP assays, multiplex Luminex technology, tetraplex immunoassays, handheld smartphone spectrometers, sensitive antibody fluorescence immunosorbent assays, pseudotype neutralization assays, and fluorescence polarization immunoassays, were developed to accurately detect antibodies against various zoonotic viruses and showed high sensitivity, specificity, and potential for field applications and vaccine quality control [94,95,96,97,98,99,100]. A series of microarray assays were developed to detect antibodies against various avian and swine pathogens with high specificity and sensitivity and with applications ranging from distinguishing AIV subtypes to serving as multi-diagnostic tools for pig sera and rapid detection methods for H9 antibodies [88,101,102,103]. A phase-intensity surface plasmon resonance (SPR) biosensor was developed for the detection of AIV A/H5N1 antibody biomarkers, with a detection limit of more than 300% better than that of commercial Biacore systems. The biosensor was also reported to be label-free and suitable for real-time monitoring and applicable for influenza detection [104].

## 4. Discussion

From 2017 to 2024, serological surveillance studies on AIV in domestic birds and backyard flocks showed circulation of AIV H5 and H9 subtypes in domestic birds, particularly chickens and ducks, in several countries globally. Domestic poultry and backyard ducks in low-biosecurity environments often served as silent reservoirs, contributing to undetected circulation and environmental contamination. Serological surveillance of AIV in domestic swine helped identify antibodies specific to AIV strains H5, H7, and H9, indicating past exposure and potential cross-species transmission events. These findings underscore the need for coordinated, cross-sectoral surveillance strategies that include both animal and human populations, especially in settings where humans and animals live in close proximity. The findings of this study also highlight the importance of enhancing biosecurity measures to minimize the risk of AIV introduction from avian sources into the swine population. More extensive serological surveillance is required to identify biosecurity gaps and inform targeted interventions. While a limited number of studies have reported serological surveillance in mammals, they have highlighted the high prevalence and cocirculation of antibodies to different subtypes (H5, H7, and H9). This underscores the potential for interspecies transmission in multi-host environments and the need for expanded serological tracking beyond traditional avian reservoirs. Additionally, many studies reported serological surveillance of swine influenza viruses aiming at identifying the risk factors for SIV seropositivity and monitoring the prevalence and spread of the virus in pig populations, as well as assessing its potential threat to human health. Studies provided evidence of reverse zoonotic transmission of IAVs from humans to pigs, further emphasizing the complexity of interspecies interactions and illustrating the bidirectional transmission of influenza and the need for joint human–animal surveillance approaches. Research into serological surveillance of canine influenza virus was sparse. Monitoring infections in companion animals is crucial considering the close contact between humans and their companion animals, interspecies transfer, and reassortment ability of influenza viruses. Serological surveillance of influenza D virus was conducted in Europe and Africa to understand the prevalence and geographical distribution of IDV, understand the disease burden in affected populations, and assess the risk for cross-species transmission.

Serological surveillance of IAV in wild animals has been less frequent but is important to understand the dynamics of the virus in its natural reservoirs and the risk of spillover events to other species. Global research efforts have focused on serological surveillance of AIV in birds between 2017 and 2024 to identify seroconversions and assess transmission risks to other species including humans. These findings support the integration of serological data with virological and syndromic surveillance to create a more complete early warning system for emerging zoonotic threats. Local serological surveillance data should be integrated with national and global influenza surveillance systems to bolster international efforts against zoonotic influenza outbreaks. Prioritizing serological surveillance in high-risk areas or populations will allow for more efficient resource allocation and rapid outbreak detection. Serological surveillances of AIV in wild mammals were conducted considering the role that animals could play in virus ecology and transmission. Studies indicate potential spillover of swine influenza viruses in wild mammalian species, highlighting the interconnectedness of these ecosystems. There are gaps in the literature, however, particularly regarding surveillance at the human–bird and wild swine–human interfaces. Sparse serological studies in these areas have identified instances where influenza viruses have crossed from birds to humans, providing insights into transmission pathways and exposure patterns. Surveillance gaps were most evident at the animal–human interface, where exposed human populations were rarely tested. Limited serological studies examining influenza viruses at the wild swine–human interface made it possible to detect cases where influenza viruses transmitted from wild swine into humans, data that could inform an assessment of potential risks for communities living near or exposed to wild swine. Designing and conducting large-scale serological studies across diverse populations and geographical areas will help collect comprehensive data on zoonotic influenza viruses.

Few serological surveillances of swine influenza virus have been conducted to understand the role of wild swine in the ecology of the virus and the impact on domestic swine and human populations since swine can act as intermediate hosts for influenza viruses and have shown the presence of antibodies against seasonal H1N1pdm virus, raising concerns of viral reassortments. Only one study reported detection of H7 antibodies, in shelter workers in New York City, suggesting transmission of influenza viruses from cats to humans. Establishing routine comprehensive serological surveillance programs for pathogens affecting domestic animals and exposed humans is essential for ongoing monitoring. These surveillance efforts should be integrated under the One Health approach to address interconnected risks.

Although new methodologies have been developed to detect antibodies against various subtypes of influenza viruses, existing ELISA methods, assay techniques, and microarrays still face technical limitations. While the novel serological methodologies offer promising advances in sensitivity, sample throughput, and field adaptability, and can enhance early detection and subtype differentiation, their adoption remains limited by several factors, including the lack of standardized protocols, dependance on specialized equipment or software, high costs, and potential limited availability of reagents in resource-limited settings. Therefore, it is crucial to develop more accurate, sensitive, cost-effective, and specific serological assays to enhance detection capabilities.

This review highlights the critical role of serological surveillance in supplementing virological monitoring, especially for the detection of subclinical and asymptomatic cases. The identification of antibodies in apparently healthy populations across species supports the need for proactive rather than reactive surveillance policies. Routine serological surveillance in high-risk animal populations and occupationally exposed human groups should be established, especially in regions with known zoonotic spillover risks, and should be integrated into national One Health surveillance platforms to support early warning systems and cross-sector risk assessments. Our findings suggest that zoonotic influenza viruses often circulate undetected in both animal and human populations. Future research should expand database searches beyond PubMed and include non-English-language studies to reduce publication bias. This review also shows that companion animals and wild mammals are not being adequately tested or included in surveillance systems, despite evidence of viral exposure, even though they may be important in the transmission or ecology of influenza viruses. Hence, surveillance of underrepresented species should be prioritized. Furthermore, access to novel and field-deployable serological assays in low- and middle-income countries should be enhanced to improve diagnostic capacity in resource-limited settings. Interdisciplinary collaboration among animal, public, and environmental health sectors is recommended to support integrated serological studies that capture the full spectrum of zoonotic influenza risk. This need for integrated surveillance aligns with the recent global consultation on H5N1 held by the WHO R&D Blueprint for Epidemics, which stress the urgency of stronger genomic surveillance, rapid data sharing, and the development of vaccines, therapeutics, and diagnostics for potential pandemic threats [105].

This review has several limitations. First, the search was restricted to a single database (PubMed) and English-language publications only, which may have excluded relevant studies published in other languages or indexed elsewhere. Second, a geographical bias might be inherent in this review and related to the fact that few countries appear to be contributing to the field of knowledge. Third, while the review spans studies from 2017 to 2024, there was considerable heterogeneity in the study designs, methodologies, and outcomes, which limited comparability and precluded formal meta-analysis. Finally, consistent with the scoping review approach, no formal quality assessment of included studies was conducted, which limits the ability to assess the robustness of included studies.

## 5. Conclusions

This scoping review mapped existing evidence on the use of serological surveillance to monitor zoonotic influenza viruses in animals and exposed human populations. The findings suggested widespread serological activity in domestic poultry and swine, with more limited data available for companion animals, wild mammals, and at the animal–human interface. A range of serological methods were identified, including several innovative technologies, although technical and implementation challenges persist. While the included studies highlighted the potential of serological surveillance to complement virological monitoring, especially for detecting asymptomatic or past infections, the review also revealed important gaps in geographic coverage, species representation, and surveillance consistency. These observations underscore the need for greater integration of serology into national and regional One Health frameworks.

This scoping review provides a foundation for identifying priority areas for future research, particularly in standardizing serological methods, expanding surveillance in understudied regions and species, and fostering multidisciplinary coordination. Strengthening serological surveillance through a One Health lens may enhance preparedness for zoonotic influenza threats. Continued research and collaboration are essential to strengthen our understanding of these pathogens and to protect both animal and human health.

## Figures and Tables

**Table 1:** Serological surveillance studies of influenza viruses in domestic birds.

Country	Species	Sample Size	Virus Tested for	Serology Test Used	Seroprevalence	Reference
Saudi Arabia	Chicken	359	AIV	Commercial ELISA kit AI (IDEXX IAV Antibody test)	12.7% *	[15]
Bangladesh	Ducks	947	AIV	Commercial cELISA kit (ID.vet ID Screen)	63.8% (95% CI: 60.6–66.8)	[16]
Bangladesh	Ducks and chicken	144 backyard, 106 broiler, and 113 layer chicken farms	AIV H5N1 and H9N2	Commercial ELISA kits (IDEXX AI MultiS-Screen ELISA; ID Screen Influenza A Antibody Competition Multi-Species ELISA; IDEXX AI ELISA)	H5: 14.2% (95% CI: 10.0–19.8%) in ducks, 4.2% (95% CI: 2.8–6.1%) in chickens H9: 15.7% (95% CI: 11.3–21.4%) in ducks, 16% (95% CI: 13.2–19.2%) in chickens	[17]
Bangladesh	Chicken	106 commercial broiler and 113 commercial layer chicken farms	AIV H5N1 and H9N2	Commercial ELISA kits (IDEXX AI, ID Screen Influenza A Antibody Competition Multi-Species ELISA)	H5: 9.4% * in broiler flocks, 3% * in layer flocks H9: 5.7% * in broiler flocks, 22% * in layer flocks	[18]
Bangladesh	Ducks	281	AIV	Commercial cELISA kit, HI assay	57.7% (95% CI: 51.6–63.3)	[19]
Brazil	Poultry	2041	AIV	Commercial ELISA kits (IDEXX Influenza A Ab Test kit, Multispecies Influenza A Antibody Test kit), and HI assay	0.7% (95% CI: 0.0–2.0) in 2016 and 0.5% (95% CI: 0.0–1.4) in 2019 in Araguaiana 0.8% (95% CI: 0.2–1.3) in 2016 and 7.0% (95% CI: 5.2–8.8) in 2019 in Cáceres	[20]
Indonesia	Ducks	245	AIV H5	HI assay	54.69% *	[21]
Polynesia	Chicken	135	AIV	Commercial ELISA kits (NP-ELISA) and HI assay	46% (95% CI: 35.1–58)	[22]
Morocco	Poultry	712	AIV H9N2	Commercial ELISA kits (ID Screen IBV Indirect ELISA kit, PROFLOK ^®^ Plus IBD Ab test kit) and HI assay	63.5% (95% CI: 53–72%)	[23]
Libya	Chicken	453	AIV H9N2	Commercial ELISA kits (X-OvO Limited) and HI assay	53.4% * and 46.8% * in Twisha and other farms	[24]
South Africa	Chicken	1007	AIV	Commercial ELISA kits AI (IDEXX IAV Antibody test)	1.8% (95% CI: 0.2−3.4%)	[25]
EU countries	Chicken	18,596	AIV	NA	43/18,596 * poultry establishments seropositive for H5 2/18,596 * poultry establishments seropositive for H7	[26]
EU countries	Chicken	24,419	AIV	NA	87/24,419 * poultry establishments seropositive for H5 22/24,419 * seropositive for H7	[27]
EU countries	Chicken	24,768	AIV	NA	46/24,768 * poultry establishments seropositive for H5 7/24,768 * seropositive for H7	[28]
EU countries	Chicken	24,290	AIV	NA	27/24,290 * poultry establishments seropositive for H5 4/24,290 * seropositive for H7	[29]
EU countries	Chicken	18,490	AIVs	NA	15/18,490 * poultry establishments seropositive for H5	[30]

CI = confidence intervals; NA = not available; * = CI not reported.

**Table 2:** Serological surveillance studies of avian influenza virus in swine and mammals.

Country	Species	Sample Size	Virus Tested For	Seroprevalence	Reference
France	Pig	10 pig herds	AIV H5	1 backyard pig in 1/10 pig herds * had antibodies against H5 clade 2.3.4.4b	[31]
Senegal	Swine	1636	AIV	83.5% (95%CI: 81.6–85.3) seroprevalence of antibodies against either H9N2, H5N1, H7N7, or H5N2	[32]
Italy	Pig	67	AIV H5N1	73% * in swine	[33]
China	Mink and fox	347 minks and 195 foxes	AIV	6.6% * and 96.2% * seropositivity rates for H7 and H9 in mink samples, 16.4% * and 10.3% * seropositivity rates for H7 and H9 in fox samples	[34]
Italy	Dog and cat	5 dogs and 1 cat	AIV H5N1	Seroconversion was detected in five * asymptomatic domestic dogs and one cat	[35]
Saudi Arabia	Camel, sheep, goat, dog, and cat	195 dromedary camels, sheep, goats, dogs, and cats	IAV	Overall IAV seropositivity rate of 4% * among unvaccinated dogs	[36]
Spain	Cat	183	IAV	2.2% (95% CI: 0.85–5.48%) seropositivity rate for anti-influenza A antibodies	[37]
Thailand	Pig	237	IAV	Absence of antibodies against IAV	[38]

CI = confidence intervals; * = CI not reported.

**Table 3:** Serological surveillance studies of swine influenza virus in swine and pigs.

Country	Species	Sample Size	Virus Tested For	Seroprevalence	Reference
Zambia	Pig	246	Human A(H1N1)pdm09	32% * in sera collected in 2011 5.3% * in sera collected in 2012 and 2018	[39]
Indonesia	Pig	649	IAV-S	26% (95% CI: 20–33)	[40]
Burkina Faso	Pig	600	Human A(H1N1)pdm09	6.8% *	[41]
China	Swine	649	IAV-S (Eurasian avian-like H1N1, pandemic H1N1, and H3N2)	IAV-S: 48.8% * Eurasian avian-like H1N1: 24.7% * Pandemic swine H1N1: 7.9% * H3N2: 0.1% *	[42]
Brazil	Pig	1631	IAV-S (H1N1 and H3N2)	IAV-S: 75% *	[43]
Brazil	Swine	233	IAV-S (H1N1, H1N2, and H3N2)	Swine H1N1: 51.9% * Codetection H1N1 and H1N2: 38.1% * H1N2: 8.6% * Codetection H1N1 and H3N2: 0.6% *	[44]
Greece	Pig	1416	IAV-S (H1N1, H1N2, and H3N2)	Vaccinated pig farms: 54% * Unvaccinated pig farms: 23% *	[45]
Serbia	Pig	222	Swine viral diseases	0%	[46]
Brazil	Pig	962	IAV-S	24.1% (95% CI: 20.71–27.55) of samples from intensive herds No positive samples in extensive rearing herds	[47]
Global	Swine	16,328	IAV	IAV: 18.28% * Human A(H1N1)pdm09: 18.92% *	[48]
Brazil	Pig	21 pig herds	IAV-S	29.2 to 51.8% * throughout the raising phases	[49]

CI = confidence intervals; * = CI not reported.

**Table 4:** Novel methods developed from 2017 to 2024 for the serological detection of antibodies against influenza viruses.

Method	Application	Technical Limitations and Implementation Challenges (When Identified)
ELISA methods		
Single M2e peptide (sM2e) ELISA [86]	Identify antibodies against AIV M2e protein	
Tetrameric M2e peptide (tM2e) ELISA [86]	Identify antibodies against AIV M2e protein	
MAb-based competitive ELISA [87]	Detect antibodies against influenza D virus	
Competitive ELISA immunoassay (cELISA) [88]	Detect antibodies against H7 based on mAb 2F8 against recombinant H7-HA1 protein	Not suitable for outbreak diagnosis
Competitive ELISA immunoassay (cELISA) [89]	Detect antibodies against H7 based on a monoclonal antibody (mAb) directed against hemagglutinin (HA) gene neutralizing epitopes	
HA1 protein-based ELISA [90]	Detect antibodies against H5	
single-domain antibodies (sdAbs) ELISA [91]	Detect antibodies against swine influenza virus	Higher false-positive rate
Nanobody-based competitive ELISA [92]	Detect anti-IAV antibodies in different species	
Epitope-blocking ELISA (H5 EB-ELISA) [93]	Detect H5 antibodies in chicken sera	Less sensitive than the commercial test in detecting anti-H5 HA antibodies in H5 influenza virus-infected chicken
Assays methods		
Duplex xMAP assay [94]	Detect antibodies against Newcastle disease virus and AIV	
Multiplex serological assay using Luminex xMAP [95]	Detect subtype AIV antibodies in poultry sera	Sophisticated equipment needed for the analysis of data
tetraplex inhibition fluorescent microsphere immunoassay (4plex iFMIA) [96]	Detect antibodies against Newcastle disease virus, AIV H5, and AIV H7 in a single serum sample	Time required to measure 96-well plates Dependency on Luminex/Bioplex machinery Lower specificity in avian field sera than HI assay
Sunlight-based handheld smartphone spectrometer [97]	Detect H7N9 antibodies	Lower sensitivity than that of the commercial microplate reader
sensitive antibody fluorescence immunosorbent assay (SAFIA) [98]	Detect H9N2 antibodies	Low detection rate
Pseudotype neutralization assay carrying H5 and H7 hemagglutinins [99]	Detect neutralizing antibodies and show specificity for H5 and H7 subtypes	
Fluorescence polarization immunoassay (FPIA) [100]	Detect antibodies against H5	Improving sensitivity is needed
Microarray methods		
Triplex protein microarray assay [88]	Detect antibodies against influenza B virus, Newcastle disease virus, and AIV	weakly positive serum samples need confirmation of results by other more sensitive serodiagnosis testing
Blocking protein microarray [101]	Detect AIV antibodies and simultaneously distinguish between H5 and H7 subtypes	
Protein microarray-based assay [102]	Simultaneously detect IgG antibodies against pathogens in pigs	
Quantitative test strips [103]	Detect antibodies against H9	

## Data Availability

All the data are present in the manuscript.
